# A primary-school-based study to reduce prevalence of childhood obesity in Catalunya (Spain) - EDAL-Educació en alimentació: study protocol for a randomised controlled trial

**DOI:** 10.1186/1745-6215-12-54

**Published:** 2011-02-27

**Authors:** Montse Giralt, Rosa Albaladejo, Lucia Tarro, David Moriña, Victoria Arija, Rosa Solà

**Affiliations:** 1 Unitat de Farmacobiologia, Universitat Roviri i Virgili, Reus Spain; 2 Educació i Promoció de la Salut, Facultat de Medicina i Ciències de la Salut, Hospital Universitari Sant Joan, Universitat Rovira i Virgili, Reus, Spain; 3 Centre Tecnologic de Nutrició i Salut (CTNS), Reus, Spain; 4 Epidemiologia Nutricional, Facultat de Medicina i Ciències de la Salut, Hospital Universitari Sant Joan, Universitat Rovira i Virgili, Reus, Spain; 5 Unitat de Recerca en Lípids i Arteriosclerosi, CIBERDEM, Hospital Universitari Sant Joan, IISPV, Universitat ROVIRA i VIRGILI, Reus, Spain

## Abstract

**Background:**

The EdAL (***Ed****ucació en ****Al****imentació*) study is a long-term, nutrition educational, primary-school-based program designed to prevent obesity by promoting a healthy lifestyle that includes dietary recommendations and physical activity.

The aims are: 1) to evaluate the effects of a 3-year school-based life-style improvement program on the prevalence of obesity in an area of north-west Mediterranean 2) To design a health-promotion program to be implemented by health-promoter agents (university students) in primary schools.

**Methods/Design:**

1) The intervention study is a randomised, controlled, school-based program performed by university-student health-promoter agents. Initial pupil enrolment was in 2006 and continued for 3 years. We considered two clusters (designated as cluster A and cluster B) as the units for randomisation. The first cluster involved 24 schools from Reus and the second involved 14 schools from surrounding towns Cambrils, Salou and Vilaseca combined in order to obtain comparable groups. There are very good communications between schools in each town, and to avoid cross influence of the programs resulting from inter-school dialogue, the towns themselves were the unit for randomisation. Data collected included name, gender, date and place of birth at the start of the program and, subsequently, weight, height, body mass index (BMI) and waist circumference every year for 3 years. Questionnaires on eating and physical activity habits are filled-in by the parents at the start and end of the study and, providing that informed consent is given, the data are analysed on the intention-to-treat basis.

The interventions are based on 8 nutritional and physical activity objectives. They are implemented by university students as part of the university curriculum in training health-promoter agents. These 8 objectives are developed in 4 educational activities/year for 3 years (a total of 12 activities; 1 h/activity) performed by the health-promoter agents in primary schools. Control pupils follow their usual activities.

2) Courses on education and promotion of health, within in the curriculum of medicine and health sciences for university students, are designed to train health-promoter agents to administer these activities in primary schools.

**Discussion:**

This controlled school-based intervention will test the possibility of preventing childhood obesity.

**Trial registration number:**

ISRCTN: ISRCTN29247645

## Background

Obesity is one of the main determinants of avoidable disease burden [1-3]. *In lieu *of affordable, non-invasive, effective obesity treatment over the long-term, and because the adverse effects of obesity on health status are not fully reversible, a stronger focus on the prevention of obesity has been advocated [4]. Since overweight status and obesity in adulthood are predicated on childhood and adolescent weight, obesity prevention should start early in life. One important target group is the child of school-age i.e. the age-group around adolescence [5]. Although genetic factors may influence the susceptibility of individuals to weight gain [6], there is a consensus that changes in lifestyle activities have driven the current obesity epidemic [7]. Therefore, obesity prevention among school children should target dietary habits as well as physical activity and sedentary behaviour [4].

The school environment is regarded as a good setting for the promotion of health interventions among children because no other institution has as much contact time with the target population [8]. Schools provide an environment where almost all children can be reached repeatedly and continuously, and where health education can be combined with health promoting environmental changes [5]. The development of personal skills and coping practices involves the promotion of healthier behaviour, particularly with respect to nutrition and physical activity, and this can be achieved by health education. Health education attempts to provide information regarding healthy diets and physical activity by means of active participation of the target population and, in soliciting a response, to promote health as the process of enabling people to increase control over, and to improve, their health [9]. Officially, teachers are preoccupied with academic activities and an option may be to involve young students from medical and health science departments of the local university who, as part of their new curriculum, receive health education oriented towards school-based interventions.

Although some school-based interventions have had positive effects on overweight and/or obesity [10-12] most, particularly those involving large cohorts [13-15], have not.

The effects of a long-term school-based intervention on improving diet and increasing physical activity and reducing obesity in the north-west Mediterranean area are, as-yet, unknown. Our hypothesis is that a regular systematic educational intervention in primary school improves lifestyle choices and reduces obesity.

As such, the aims of the study are: 1) To evaluate the effects of a 3-year school-based program of lifestyle improvement, including diet and physical activity, on the prevalence of obesity; 2) To design a health promotion program for implementation by university students acting as "health promoting agents" (HPA) in primary schools.

## Methods/Design

### University program

The training focuses on promoting a healthy lifestyle via activities designed to reduce obesity, and to prepare the HPA to implement these educational interventions in schools.

The intervention approach combines constructs from education- and nutrition-based evidence.

Two courses are proposed:

Course #1: Methodology for the promotion of health in schools

Description of the course

Bases of health education and health behaviour

Theory and program planning: A study of determinants of health behaviour, factors influencing health behaviour, health behaviour theories and application of methodology are examined.

Health education curriculum and instruction: Research methods in health education; principles of evidence-based nutrition.

12 lectures of 1 h duration each, delivered over 15 weeks per academic year. Eight topics in nutrition are chosen for scientific evidence to improve consumption of some foods. Increased physical activity and healthy habits such as teeth-brushing and hand-washing are highlighted.

The objectives are:

1. Healthy lifestyle. Taste (knowledge of previously-unknown food items)

2. Healthy drinks

3. Vegetables and legumes

4. Candies and pastry *vs*. nuts

5. Healthy habits: timetable (home meals preferably) and physical activity

6. Fruits

7. Dairy products

8. Fish

Course #2. Interdisciplinary implementation of health education in schools

This course provides a careful examination of strategies of design, implementation, and health program evaluations, including training and standardisation of each activity as well as the performance of these activities in schools.

In the activity training, the primary school teachers are included in the jury to assess the performance of the HPA in the primary schools.

4 lectures of 1 h duration each; in 12 h for training and standardisation and 12 activities (1 h/activity/classroom) over 15 weeks per academic year

### Schools

The school-based lifestyle modification program is designed as an interdisciplinary health promotion program performed by HPA.

All strategies are focused on children aged between 7 and 8 years. The program targets the whole school community including parents, pupils, staff and teachers within the school environment. The schools for the program needed to be representative of the child population. We offered the program to all schools whether public (funded by the government and termed "charter" schools) or private. To maintain independence between intervention and control schools, we selected schools from Reus (a town with about 100,000 citizens) to represent the intervention group, with Cambrils, Salou and Vilaseca (3 towns on the outskirts of Reus with about 70,000 citizens in total) to represent the control group. There are very good communications between schools within each town and, hence, to avoid cross influence between control schools that were masked with respect to intervention, the towns themselves were the units for randomisation.

The coordinating Centre developed a randomization scheme in which the schools in Reus (designated as group A) and the schools in the three others towns of Cambrils, Salou and Vilaseca (designated as group B) had similar pupil populations.

Randomisation defined A group as the intervention and B as the control group.

In our area of Spain, each classroom contains approximately 25 children (at most 27 children and, occasionally, 20-22 children). When the numbers of children exceed the state-recommended level, a new classroom at the same education level is opened in the school. Thus, there could be 2 or more classrooms for any specific level; some schools may even have 3 classrooms per level with 25-27 children per classroom. All classes are co-educational

Participating intervention institutions consist of 24 schools involving 36 classrooms (97% public or state funded "charter" schools and 3% private) involving at least 700 pupils in Reus. Participating control institutions consist of 14 schools involving 39 classrooms (96% public or state funded "charter" and 4% private) involving at least 700 pupils in the 3 surrounding towns of Cambrils, Salou and Vilaseca. The ethnic origin of control and intervention pupils are: 83.2% of Western European descent (mainly from Catalunya) and 16.8% are non- European; 7.8% from Latin America, 5% of North-African Arab descent, 3% recent immigrants from Eastern Europe (mainly Rumanian) and from other countries from the Asian Far East (such as China).

For a school to be included in the study, at least 50% of the children in the classrooms needed to have volunteered to participate. In the intervention group, all children of the selected classroom are exposed to the intervention. For logistics reasons, the first scholastic group was enrolled in 2006 and followed-up for 3 years (2006-2009), and a second scholastic group was enrolled in 2007 and also followed-up for 3 years. Thus the final measures are performed in the year 2010. The data are collected on all the children, but only the data from individuals (and their parents) who provided informed consent are included in the final analyses.

Name, gender, date and place of birth are recorded at the start of the program, while weight, height, body mass index (BMI) and waist circumference variables (identified set of anthropometric measures) are recorded in each of the 3 years of the study. The measurements were performed in May of the years 2006, 2007, 2008, 2009, and 2010.

Body weight was measured to the nearest 0.05 kg using a standard beam balance (Tanita TBF-300 Body Composition Analyzer, Brooklyn NY, USA). The set of anthropometric measurements was performed three times each academic year. The mean values were used in all subsequent statistical analyses.

To assess intra-observer variability, the anthropometric measurements were repeated in 20 children (10 boys and 10 girls). To assess inter-observer variability, all 5 observers conducted the 5 anthropometric measurements in 10 children. The standardisation of observers was performed in each year of the study [16].

If the child is lost to follow-up (the child is relocated to a different school, or the parents move to a different area) we are able to contact the child/parents via the name and date of birth; data that are held in the records of the local education authority. If the child moves to a school that is participating in the study, he/she is identified and followed-up within the new school. If, instead, the children move to another city outside the study area, they are lost to the study and are not replaced. We anticipate a loss of about 20% of pupils which, by chance, would be similar in intervention and control schools. The assumption would be that all losses are, also, by chance and the statistical methods used are robust enough to accommodate for randomly-produced missing values.

Questionnaires regarding eating habits (Krece-Plus) developed by Serra-Majem et al [17] and physical activity as well as the level of parental education and their habits [18] are filled-in by the parents at the start and conclusion of the study.

### Intervention program

The intervention program consisted of three components:

1. Classroom practice by HPA to highlight healthy lifestyle habits

2. Teaching practice by HPA using books designed to include the nutritional objectives

3. Parental activities included with their children

In each of 12 activities (1 h/activity), the classroom practice consisted of three components:

1. Experimental development of activities regarding each healthy lifestyle habit

2. Assessment of activity performed in classroom

3. An activity developed for use at home

### Approval

This study was approved by the Clinical Research Ethical Committee of the Hospital Universitari Sant Joan of Reus, Universitat Rovira i Virgili (Catalan ethical committee registry #20; ref: 08-07-24/07aclproj1). The protocol conformed to the Helsinki Declaration and Good Clinical Practice guides of the International Conference of Harmonization (ICH GCP). This trial is registered with International Standard Randomised Controlled Trial Register, number ISRCTN29247645.

### Statistical Analyses

We estimated that with a sample of 700 pupils per group, the study would have 83.5% power to detect a difference of 5 percentage points between the intervention and control schools (9%-14%), with respect to the primary outcome (prevalence of obesity), setting the bilateral level of statistical significance at 5%.

Descriptive data are presented as means ±SD (95%CI) or percentages. Generalised linear mixed models were used to analyse differences between the intervention and control pupils with respect to the primary outcome.

Measurements were conducted at baseline when the pupils are 2^nd^-3^rd ^graders (6 or 7 year olds) and at the end of the study when they are 4^th^-5^th ^graders (9 or 10 year olds).

Primary outcomes include obesity (BMI ≥95^th ^percentile) and overweight (BMI ≥85^th ^percentile) based on the 1988 BMI tables of Hernandez [19]. We analysed obesity and overweight and a measure of thinness according to the Cole criteria [20,21] as well as other measures of adiposity such as BMI z score and waist circumference. The numbers of subjects having a particular dietary habit are expressed as percentages of the total number of individuals being evaluated.

## Discussion

Developing personal skills and coping practices involving the promotion of healthier behaviours or habits can be induced by health education, particularly with respect to nutrition and physical activity. Nutrition and health education programs attempt to inculcate nutritional values or healthy diets by means of active participation of the target population, often involving person-to-person interactions.

Although genetic factors may influence the susceptibility of individuals to weight gain [6], there is growing awareness that recent changes in lifestyle habits underlie the current obesity epidemic [7].

We chose BMI as the measure of obesity and the changes in BMI as the effectiveness of the education program. Although waist circumference (WC) or skin-fold thickness, particularly tricipital, or BMI z score can be used as the measure of obesity, BMI values are acceptable.

We proposed 3 evaluation methods based on the tables of Hernández et al and Cole et al [18,19] and the EnKid study of 2003 [22] because each one has advantages and inconveniences. Hernandez is the oldest, and the values of BMI are the lowest corresponding to data before the increase in prevalence of overweight and obesity. The Enkid study contains BMI values from 1998-2000, which are ten years more recent than that of Hernández [18], and describe the increase in BMI in a youth population. Obesity is defined as BMI ≥95^th ^percentile and overweight by BMI ≥85^th ^percentile in order to compare the results of United States guidelines [23]. The tables from Cole et al [19,20] enable comparisons to be made with European studies.

We standardised intra- and inter-observer variation to assure quality in the anthropometric measurements.

The results expected are the reduction of obesity prevalence based on improving lifestyle habits. Wang et al [24] had proposed that 1% increase/year in childhood obesity in Mediterranean Europe would induce an estimated 11.5% obesity and 30.2% overweight prevalence by the year 2010 and, as such, increase in prevalence of obesity and overweight will have an important health effect.

The lifestyle interventions in the program implemented by the university students being trained as HPA include experimental activities using natural food in the habitual diet, and increasing physical activity in the schools. Multi-component foods, physical education, class curricula, behavioural knowledge and skills, communications and social marketing, and the acceptability of healthy behaviour have been proposed as means of reducing BMI. However, the efficacy observed has been inconsistent. Evidence for effectiveness on anthropometrical obesity-related measures is lacking [25].

The challenge is to identify and to develop a cohesive hypothesis which combines educational and health promotion, to examine the effects of the intervention with valid and reliable measurements of the outcomes.

## Competing interests

The authors declare that they have no competing interests.

## Authors' contributions

MG participated in the conception and design of the study and its final approval, drafting and revising the manuscript. RA participated in designing the study, drafting and revising the manuscript. DM participated in designing the biostatistical methods of the study, drafting and revising the manuscript. RS participated in designing the study, drafting and revising the manuscript.
